# Independent Association of Waist Circumference With Hypertension and Diabetes in African American Women, South Carolina, 2007–2009

**DOI:** 10.5888/pcd9.110170

**Published:** 2012-05-24

**Authors:** Tatiana Y. Warren, Sara Wilcox, Marsha Dowda, Meghan Baruth

**Affiliations:** Author Affiliations: Sara Wilcox, Marsha Dowda, Meghan Baruth, University of South Carolina, Columbia, South Carolina.

## Abstract

**Introduction:**

Obesity is associated with hypertension and diabetes, which are independent risk factors for cardiovascular disease (CVD); 53% of African American women are obese. Of the approximately 44% of African American women who are hypertensive, more than 87% are overweight or obese. Additionally, more than twice as many African American women (13.1%) as white women (6.1%) have been diagnosed with type 2 diabetes. Obesity is usually measured using body mass index (BMI). However, abdominal adiposity may be more predictive of CVD risk than BMI. This study investigates the independent association of waist circumference with hypertension and diabetes in African American women.

**Methods:**

As part of the Faith, Activity, and Nutrition (FAN) program, we recruited 843 African American women (mean age 53.8 y [SD, 14.1 y]) from African Methodist Episcopal churches. If a participant reported she had hypertension or had measured systolic blood pressure at or higher than 140 mm Hg or measured diastolic blood pressure at or higher than 90 mm Hg, she was classified as having hypertension. To assess increased health risks associated with waist circumference, we used the World Health Organization’s standards to categorize waist circumference as normal risk (waist circumference <80 cm), increased risk (waist circumference 80–88 cm), or substantially increased risk (waist circumference >88 cm). We used logistic regression models to test predictors of hypertension and diabetes.

**Results:**

Of 843 study participants, 205 had diabetes and 545 were hypertensive. Women with a waist circumference of 88 cm or more were at increased risk for hypertension (odds ratio [OR] = 7.17, *P* < .002) and diabetes (OR = 6.99, *P* < .001). Associations remained after controlling for all variables (hypertension OR = 5.53, *P* < .001; diabetes, OR = 5.38, *P* < .001).

**Conclusion:**

After controlling for all variables, waist circumference was independently associated with a 5-fold risk in hypertension and diabetes in African American women.

## Introduction

Obesity is associated with hypertension and diabetes, which are risk factors for cardiovascular disease (CVD) (1). Fifty-three percent of African American women are obese (2). Recent reports indicate that approximately 44% of African American women are hypertensive (2), and of these, more than 87% are overweight or obese (1). Additionally, national data show that the prevalence of diagnosed type 2 diabetes is more than twice as high among African American women (13.1%) as among white women (6.1%) (3). Abdominal adiposity is a risk factor for obesity-related complications, and there is increasing evidence that abdominal adiposity may be a contributing factor to complications not related to adiposity at the waist (4,5).

Body mass index (BMI), waist-to-hip ratio, and waist circumference are commonly used measures for estimating abdominal adiposity (6,7). BMI is a simple and widely used clinical measure; however, BMI may not be a reliable indicator of health risk across all racial and ethnic groups (6,8,9). This may be due, at least in part, to errors inherent in the use of self-report measures of BMI (6). Considerable attention has been given to waist circumference as a complementary (10) and, in some cases, superior (8) assessment to BMI.

Waist circumference is a practical method for assessing CVD risk factors in whites (11); however, few studies have examined the association between waist circumference and risk factors in African Americans (9,12,13). Our study objective was to examine the independent association of waist circumference with hypertension and diabetes in African American women. We hypothesize that the risk of hypertension and diabetes would be higher for women with larger waist circumference, independent of sociodemographic and health-related variables.

## Methods

Faith, Activity, and Nutrition (FAN) is a 5-year, faith-based study. It represents a university-church partnership to promote physical activity and healthy eating among members of African Methodist Episcopal (AME) churches. The primary goals of FAN are to increase physical activity, increase fruit and vegetable consumption, and improve blood pressure among its participants. Secondary goals are to increase participants’ consumption of whole grains and decrease their sodium and fat consumption. Our study was part of the overall FAN study.

### Research design

FAN uses a randomized design with a delayed-intervention control group and takes place in 3 waves (14). Each wave lasts approximately 30 months and completes an intervention cycle, 15 months for churches randomized to early intervention and 15 months for churches receiving the delayed intervention. Outcome measures are taken at baseline and at post intervention. At the end of the 15-month intervention, delayed-intervention churches have an opportunity to implement the FAN program, but no further follow-up occurs. Details of the overall intervention study are described elsewhere (14). Our study uses baseline data from the larger FAN study (2007 through 2009) and thus is a cross-sectional design.

### Church and participant recruitment

Presiding elders of 4 geographically defined districts in South Carolina (Kingstree, Georgetown, Columbia, and Mount Pleasant) sent a letter introducing the FAN program to pastors in their districts. Interested churches were asked to complete and return a contact information form to FAN staff, who then made follow-up telephone calls to pastors to address questions or concerns. Churches agreeing to participate in FAN were asked to sign a memorandum of agreement. Pastors usually designated the church health director or another church leader to serve as FAN coordinator. This person acted as the liaison between the church and FAN staff in recruiting members of their congregation to take part in a measurement session. Small churches were asked to recruit at least 13 members; medium churches, 32 members; and large churches, 63 members. More recruitment details are provided elsewhere (14).

### Measurement/data collection

We recruited a total of 843 African American women from AME churches in the 4 targeted districts. Written informed consent was obtained from all participants, and our study was approved by the institutional review board of the University of South Carolina. We included only women in our study because of the small percentage of men participating in FAN. Trained study staff measured participants’ height, weight, waist circumference, and blood pressure. Participants completed a survey assessing sociodemographic characteristics (age, marital status, and education), physical activity levels, diet, and general health. To be eligible for our study, participants had to be at least 18 years of age and free of serious medical conditions or disabilities that would make physical activity difficult. They had to attend worship services at least once a month and plan to reside in the area for the next 2 years. These criteria were presented in the informed consent form; therefore, nonqualifying participants were self-excluded.

### Measures

Participants removed shoes, excess clothing, and all items in their pockets before having their height and weight measured. A Seca 770 digital scale (Seca Corporation, Hanover, Maryland) measured weight to the nearest tenth of a kilogram, and a Seca stadiometer (Seca Corporation, Hanover, Maryland) measured height to the nearest quarter of an inch. We calculated BMI by dividing weight in kilograms by height in meters squared. Participants were categorized as not obese (BMI <30) or obese (BMI ≥30). Because the BMI of very few participants was in the normal weight category, the normal weight and overweight groups were combined in analyses and defined as not obese.

Participants were asked to remove all excess clothing before we measured waist circumference. With the woman standing upright, we measured waist circumference at the narrowest part of the participant’s torso (or the minimum circumference between the rib cage and the iliac crest) (15) using an anthropometric measuring tape. The measurement was taken at the end of expiration. We measured waist circumference, recorded to the nearest tenth of a centimeter, 2 to 3 times and used the average of the 2 closest measurements (within 2 cm). Participants were categorized as normal risk (<80 cm), increased risk (80–88 cm), or substantially increased risk (>88 cm) on the basis of the World Health Organization’s standards for increased health risk associated with waist circumference (16).

Before measuring blood pressure, we asked participants to sit quietly for 5 minutes with legs uncrossed and to remove any excess clothing. We used an automated DinaMap ProCare 100 monitor (Critikon, Inc, Tampa, Florida) to measure seated blood pressure. We placed the blood pressure cuff on the participant’s upper right arm at heart level and repeated the measurement 3 times with 30 seconds of rest between measurements. We used the average of the second and third readings. Hypertension was classified as mean systolic blood pressure at or higher than 140 mm Hg, mean diastolic blood pressure at or higher than 90 mm Hg, or self-reported hypertension. Self-reported hypertension was assessed with the question, “Have you ever been told by a doctor, nurse, or other health professional that you have high blood pressure?”

To assess diabetes, we asked participants if they had ever been told by a doctor, nurse, or other health professional that they had diabetes. Women who answered “yes” or “yes, but only during pregnancy” (n = 9) were classified as having diabetes.

To measure physical activity, we used a modified version (36-item vs 41-item measure) of the Community Health Activities Model Program for Seniors (CHAMPS) questionnaire (17,18). We excluded 5 items from the questionnaire to make the process less burdensome for participants and added a single item measuring frequency of dancing and moving during church services. This version is similar to the CHAMPS questionnaire described by Resnicow et al (18). We asked participants to self-report the frequency and duration of various physical activities that they completed “in a typical week during the past 4 weeks.” Included were activities typically undertaken for exercise (eg, walking, yoga, aerobics), physical activities undertaken in the course of one’s daily routine (eg, housework, yard work, other daily activities), and recreational activities that provide physical activity (eg, tennis, golf). Hours per week spent in light-, moderate-, and vigorous-intensity physical activity (ie, all activities with metabolic equivalent values of 2.0 or greater) were then computed to determine mean hours per week of total physical activity. Duration was assessed as the number of times per week using a 6-item scale that ranges from “less than 1 hour per week” to “9 or more hours per week.” CHAMPS has demonstrated strong psychometric properties, including test-retest reliability and validity (17) and sensitivity to change (19).

The National Cancer Institute fruit and vegetable 9-item all-day screener was used to assess participants’ daily fruit and vegetable consumption (in cups). This scale correlates moderately with 24-hour recall measures of fruit and vegetable consumption (20), which are considered the gold standard in dietary research (21).

### Statistical analyses

We used SAS statistical software (version 9.2, SAS Institute, Inc, Cary, North Carolina) to analyze data. Frequencies, means, and standard deviations were calculated for self-report, demographic, and health-related behaviors. Total physical activity was positively skewed at baseline but was normalized with a square-root transformation. Logistic regression analyses were used to examine the associations between waist circumference (independent variable, defined as normal, increased risk, or substantially increased risk) and both diabetes and hypertension (dependent variables). Because analyses that accounted for dependency among participants from the same church (ie, participants nested within churches) did not differ from those that did not account for this dependency, the latter are presented because of their simplicity in interpretation.

Multivariate analysis was conducted to control for numerous potential confounding variables known to influence each of the dependent variables. Model 1 included only waist circumference. To control for potential sociodemographic confounding variables that may be associated with hypertension and diabetes, adjustments for age (continuous), education (less than high school graduate or high school graduate, some college or more), and marital status (married, not married) were included in model 2. Model 3 adjusted for health-related variables: smoking (smoker, nonsmoker), BMI (normal weight, overweight, and obese), physical activity (continuous hrs/wk), and fruit and vegetable consumption (cups/day). The final adjusted model (model 4) included waist circumference and all sociodemographic and health-related variables.

## Results

At baseline, a total of 971 women participated in FAN; we excluded 128 of these women from our study because data were missing on 1 or more variables needed for analyses. Data on 843 participants were included in the analysis (Table 1). Most participants had at least some college education (60.6%), and approximately half reported being married (49.7%). Mean age was 53.6 years (SD, 13.8 y), and mean BMI was 33.6 kg/m^2^ (SD 7.6 kg/m^2^). Twenty-eight percent of participants were overweight and 66% were obese. There was a strong positive correlation between BMI and waist circumference (*r* = 0.821, *P* < .01) (data not shown). Of the total sample, 70% of the participants had a waist circumference associated with a substantially increased health risk (>88 cm). Most study participants had hypertension (64.6% self-reported hypertension or had measured hypertension) and a quarter (24.6%) reported having diabetes. Less than 6% of study participants reported currently smoking. On average, participants consumed 3.7 cups of fruits and vegetables per day and participated in 13.2 hours of total physical activity per week (data not shown).

**Table 1:** Baseline Characteristics of Participants (N = 843), Faith, Activity, and Nutrition Study, South Carolina, 2007–2009

Characteristic	Value^a^
**Age, mean (SD), y**	53.6 (13.8)
**Body mass index, kg/m^2^**	
All participants, mean (SD)	33.6 (7.6)
Normal weight (<25.0)	52 (6.2)
Overweight (25.0–29.9)	236 (28.0)
Obese (≥30)	555 (65.8)
**Smoking status**	
Smoker	50 (5.9)
Nonsmoker	793 (94.1)
**Fruit and vegetable consumption, mean (SD), cups/d**	3.7 (3.5)
**Physical activity,^b^ mean (SD), h/wk^a^**	13.2 (10.7)
**Education**	
≤High school graduate	332 (39.4)
Some college or more	511 (60.6)
**Marital status**	
Married	419 (49.7)
Not married	424 (50.3)
**Hypertension**	
No hypertension	298 (35.4)
Hypertension	545 (64.6)
Self-reported but not measured	264 (31.3)
Self-reported and measured	189 (22.4)
Measured but not self-reported	92 (10.9)
**Diabetes**	
No diabetes	628 (75.4)
Diabetes only during pregnancy	9 (1.1)
Has diabetes	205 (24.6)
**Waist circumference, cm**	
Normal (<80)	87 (10.3)
Increased (80–88)	166 (19.7)
Substantially increased (>88)	590 (70.0)

Abbreviations: SD, standard deviation; BMI, body mass index.

^a^ Values expressed as percentages, unless otherwise noted.

^b^ Total physical activity time spent in light-, moderate- and vigorous-intensity physical activity. Includes activities typically undertaken for exercise (eg, walking, yoga, aerobics), physical activities undertaken in the course of one’s daily routine (ie, housework, yard work, and other daily activities) and recreational activities that provide physical activity (eg, tennis, golf).

### Hypertension

In model 1, increased-risk waist circumference and substantially increased-risk waist circumference were independently and significantly associated with hypertension (*P* < .002) (Table 2). After controlling for sociodemographic variables in model 2 (*P* < .001) and health-related variables in model 3 (*P* = .02), the association between waist circumference and hypertension remained significant. After controlling for all sociodemographic and health-related variables in model 4, increased-risk waist circumference and substantially increased-risk waist circumference remained independently and significantly associated with an increased risk for hypertension (*P* < .001).

**Table 2:** Association of Waist Circumference with Hypertension in African American Women, Faith, Activity, and Nutrition Study, South Carolina, 2007–2009

Waist Circumference Risk^a^	OR (95% CI)	*P* Value
**Model 1^b^**		
Normal	1 [Reference]	<.002
Increased	2.93 (1.67–5.16)	
Substantially increased	7.17 (4.31–11.93)	
**Model 2**		
Normal	1 [Reference]	<.001
Increased	2.95 (1.53–5.67)	
Substantially increased	7.03 (3.87- 2.77)	
**Model 3**		
Normal	1 [Reference]	.02
Increased	3.03 (1.71–5.36)	
Substantially increased	7.34 (3.92–13.74)	
**Model 4**		
Normal	1 [Reference]	<.001
Increased	2.79 (1.44–5.41)	
Substantially increased	5.53 (2.66–11.48)	

Abbreviations: OR, odds ratio; CI, confidence interval.

^a^ Waist circumference risk defined according to World Health Organization guidelines (16): normal risk, <80 cm; increased risk, 80–88 cm; and substantially increased risk, >88 cm.

^b^ Model 1 is unadjusted; model 2 is adjusted for age, education, and marital status; model 3 is adjusted for smoking, body mass index, total daily physical activity, and total daily fruit and vegetable consumption; and model 4 is adjusted for all variables.

### Diabetes

There was a significant relationship between waist circumference and prevalence of diabetes in model 1 (*P* < .001) (Table 3). Increased-risk and substantially increased-risk waist circumference both showed positive associations with diabetes. After we controlled for sociodemographic variables in model 2 (*P* < .001) and health-related variables in model 3 (*P* < .001), increased-risk waist circumference and substantially increased-risk waist circumference remained significantly associated with diabetes. In model 3, the odds of having diabetes were significantly greater for those with a substantially increased-risk waist circumference than for those with an increased-risk waist circumference. After controlling for all sociodemographic and health-related variables in model 4, both waist circumference categories remained significantly associated with diabetes (*P* < .001.

**Table 3:** Association of Waist Circumference with Diabetes in African American Women, Faith, Activity, and Nutrition Study, South Carolina, 2007–2009

Waist Circumference Risk^a^	OR (95% CI)	*P* Value
**Model 1^b^**		
Normal	1[Reference]	<.001
Increased Risk	3.49 (1.30–9.34)	
Substantially Increased Risk	6.99 (2.79–17.54)	
**Model 2**		
Normal	1[Reference]	<.001
Increased risk	3.10 (1.15–8.40)	
Substantially increased risk	5.53 (2.18–13.89)	
**Model 3**		
Normal	1[Reference]	<.001
Increased risk	1.24 (0.82–1.87)	
Substantially increased risk	7.40 (2.73–20.0)	
**Model 4**		
Normal	1[Reference]	<.001
Increased risk	3.25 (1.19–8.88)	
Substantially increased risk	5.38 (1.94–14.71)	

Abbreviations: OR, odds ratio; CI, confidence interval.

^a^ Waist circumference risk defined according to World Health Organization guidelines (16): normal risk, <80 cm; increased risk, 80–88 cm; and substantially increased risk, >88 cm.

^b^ Model 1 is unadjusted; model 2 is adjusted for age, education, and marital status; model 3 is adjusted for smoking, body mass index, total daily physical activity, and total daily fruit and vegetable consumption; and model 4 is adjusted for all variables.

## Discussion

Nationally, the prevalence of hypertension and diabetes is highest among African Americans (2), who are also at greatest risk for illness and death related to diabetes (2). African American women in particular have the highest prevalence of hypertension in the United States compared with all other racial and ethnic groups of both sexes, which further increases their risk of illness and death from CVD (2). Additionally, the group most disproportionately affected by obesity is African American women (2). Because a large percentage of African American women in the United States are overweight or obese and because a large percentage of these women have hypertension or diabetes, research needs to focus on the public health effect of increased abdominal adiposity.

One study suggests that the relationship between BMI and hypertension or BMI and diabetes is weaker for African Americans than for other racial groups (12). Despite this finding, there is limited research regarding the use of waist circumference measurements to assess increased risk for diabetes and hypertension in African American women. In addition, approximately 70% of women in the United States aged 50 to 79 now exceed the substantially increased risk waist circumference of larger than 88 cm (22). Data from the National Health and Nutrition Examination Survey further show that African American women have most recently (past 5 years) had the greatest increase (6.3%) in waist circumference and in the prevalence of abdominal obesity (22). This clinically defined waist circumference threshold for abdominal obesity has been associated, independent of BMI, with hypertension (23-25) and type 2 diabetes (24-26) in predominantly white populations; however, in some minority populations a J-shaped relationship has been reported (27) and, in some cases, no association (28).

Participants in our study had high rates of hypertension (65%), obesity (66%), and substantially increased-risk waist circumference (70%). Additionally, compared with all African American women in South Carolina (based on South Carolina Behavioral Risk Factor Surveillance System data), our study participants had higher rates of hypertension (64.6% vs 36.6%) and obesity (66% vs 45.4%); this was perhaps due in part to the older average age of our population (29). In our sample, increased waist circumference was also associated with higher risk of hypertension and diabetes independent of BMI. Despite the high correlation between BMI and waist circumference, a major finding was that after controlling for sociodemographic and health-related variables, including BMI, an independent and significant association between waist circumference and both diabetes and hypertension remained. Thus, waist circumference still explains variance in diabetes and hypertension that BMI does not.

Our results are consistent with previous findings. Okosun et al (9) assessed the association of waist circumference and risk of hypertension and type 2 diabetes in populations from several different African origins. Findings from this cross-sectional study showed that waist circumference was significantly and positively associated with blood pressure and fasting blood glucose, regardless of origin (9). Results further showed that participants in the highest waist circumference quartile (116.4 cm; SD, 9.8 cm) had a 2-fold increased risk for hypertension and 23-fold increased risk for diabetes compared with participants in the lowest quartile (75.9 cm; SD, 5.5 cm) (9). Participants with substantially increased waist circumference (>88 cm) had a 5-fold increase for hypertension and diabetes compared with participants with a normal waist circumference (<80 cm), adjusting for all other variables.

Studies have shown that abdominal adiposity has adverse effects on health, regardless of BMI (22). There is also a strong positive correlation between central obesity (ie, waist circumference >88 cm) and CVD (4). Furthermore, in the last decade, it appears there have been greater increases in the prevalence of abdominal adiposity among African American women (29). A major public health concern in the United States is the reduction of racial health disparities (29). CVD and associated risk factors (ie, obesity, hypertension, diabetes, and physical inactivity) disproportionately affect the lives of African American women (2). The prevalence of CVD-associated risk factors was high in our study.

A large percentage (53%) of African American women in the United States are overweight or obese (2), and a large percentage (>80%) of these women have hypertension or diabetes (1,3). Public health goals and objectives must be clearly defined to target the health effect of increased abdominal adiposity and health risk factors, especially among African American women. Measurements of waist circumference can be useful in the assessment of abdominal obesity and disease risk (12). Traditionally, BMI has been used to determine obesity and categorize persons into weight categories that may be associated with greater health risk. However, a normal BMI does not necessarily indicate normal levels of abdominal adiposity (30). Waist circumference should be considered a practical method for assessing risk factors for CVD in African American women, as it has been shown to be for white women (11,25). The use of BMI and waist circumference together could enable better assessment of individual health risks.

This study has several strengths, including the use of objective measures of waist circumference, BMI, and blood pressure. Data from our large sample of African American women add to the existing body of information on this population, which bears the greatest disease risk (2,3). Our study also has limitations. Both diabetes and physical activity were self-reported. In addition, the study used a cross-sectional design, which prevents causal inferences.

Our study results suggest a relationship between waist circumference and CVD risk factors, specifically hypertension and diabetes. One must be careful in making causal attributions from a cross-sectional study, but our results suggest that independent of BMI, maintaining a normal waist circumference (<80 cm) may reduce health risks. The attributable risk associated with substantially increased waist circumference suggests that risk of developing hypertension and diabetes could be reduced considerably by reducing waist circumference to 80 cm in African American women; however, more research is needed in this area. Suggestions for future research include 1) using a prospective design to examine the degree to which waist circumference is predictive of disease incidence, 2) comparing the predictive ability of BMI versus waist circumference in prospective studies, 3) examining whether reductions in waist circumference predict reductions in health risk over time, and 4) examining differences between men and women in the relationship between waist circumference and hypertension and diabetes.
